# 2,3,5,4'-tetrahydoxystilbene-2-O-β-D-glucoside eliminates staurosporine-induced cytotoxicity by restoring BDNF-TrkB/Akt signaling axis

**DOI:** 10.7150/ijms.47919

**Published:** 2020-08-19

**Authors:** Teng-Teng Ren, Sheng-Rui Fan, Xiu-Yuan Lang, Yun Yu, Rongfeng Lan, Xiao-Yan Qin

**Affiliations:** 1 Center on Translational Neuroscience, College of Life and Environmental Sciences, Minzu University of China, Beijing 100081, China.; 2 Department of Cell Biology & Medical Genetics, School of Basic Medical Sciences, Shenzhen University Health Science Center, Shenzhen 518060, China.

**Keywords:** Akt, BDNF, K252a, LY294002, staurosporine, THSG, TrkB

## Abstract

2,3,5,4'-Tetrahydroxystilbene-2-O-β-d-glucoside (THSG) is the major active ingredient in *Plygonum multiflorum* that displays a great deal of health-benefits including anti-oxidation, anti-hyperlipidemia, anti-cancer, anti-inflammation and neuroprotection. However, it is unclear whether THSG exerts neuroprotective functions by regulating neurotrophic factors and their associated signaling pathways. In this study, hippocampal neurons were challenged with staurosporine (STS) to establish a neural damage model. We found that STS-induced cytotoxicity introduced significant morphological collapse and initiating cell apoptosis, along with the down regulation of BDNF and TrkB/Akt signaling axis. In contrast, neurons pretreated with THSG showed resistance to STS-induced toxicity and maintained cell survival. THSG rescued STS induced dysfunctions of BDNF and its associated TrkB/Akt signaling, and restored the expression of Bcl-2 and Caspase-3. However, inhibition of TrkB activity by K252a or Akt signaling by LY294002 abolished the neuroprotective effects of THSG. Therefore, BDNF and TrkB/Akt signaling axis is a promise target for THSG mediated neuroprotective functions.

## Introduction

*Polygonum multiflorum*, also known as *He shou wu,* is a well-known Chinese herb that used as a tonic and anti-aging medicine for centuries. Pharmacologic studies have identified its components and their activities in anti-inflammation, anti-oxidation, and ameliorating sleep, etc. 1-4. In particular, 2,3,5,4'-tetrahydoxystilbene-2-O-β-D-glucoside (THSG) is the characteristic chemical and has been well established for its functions in promoting longevity, anti-depression, attenuating inflammation, anti-aging and aging related diseases 4, 5. Thus, the neuroprotective effects of THSG have been recognized increasingly. In the central nervous system, inappropriate cell death resulting from toxins, injury, oxidative stress, infectious agents or genetic diseases are the origin of series types of neural diseases. For instance, the parkinsonian toxin 1-methyl-4-phenylpyridinium induced cytotoxicity and nigrostriatal degeneration was used to establish the mouse models of Parkinson's disease (PD) 6. Accordingly, THSG is effective to ameliorate the neural death in PD mice, possibly through restoring the expression of brain-derived neurotrophic factor (BDNF) and its associated TrkB/Akt signaling pathway 7. We and colleagues reported the role of BDNF in THSG mediated anti-depressant-like effects in mild stress induced depressive mice 5, 8. Recovery the expression of BDNF may be a potential strategy for therapy of depression, schizophrenia, AD and PD, etc. 9, 10.

Staurosporine (STS) is a cell-permeable alkaloid of the indolocarbazole. It is widely used in the establishment of cell apoptosis and death model, because of its broad-spectrum and effective inhibitory activity on protein kinases and associated signaling pathways 11-13. One way of STS induced apoptosis may be mediated by activating of Caspase-3 14. In neurons, STS-induced toxicity results in severe morphological collapse and cell death, along with significant changes in gene expression. Besides, neurotrophins and their signaling effectors were most vulnerable. BDNF, for example, the most focused factor in the central nervous system, is essential for neural survival and plasticity 10, 15. Dysfunctions of BDNF and its associated signaling regulators such as TrkB, PI3K/Akt, Erk1/2 are intensively reported in the progress of neural atrophy and cell loss, which is the origin of neurodegenerative diseases. Therefore, the effect of STS on the expression and activity of BDNF and the restoration of BNDF function is important for neuropharmacological research and drug discovery 11, 16.

In this work, we examined the neuroprotective activity of THSG in cultured rat hippocampal neurons challenged by STS-induced toxicity. In the experiments, STS was administrated to produce cytotoxicity and initiate apoptosis 12. Then, THSG was added to monitor its protective effects on neurons from STS-induced toxicity. Moreover, the expression of BDNF, TrkB and Akt as well as the apoptosis-related proteins Bcl-2 and Caspase-3 were examined, to study the regulatory role of BDNF and its downstream signaling axis in THSG mediated neuroprotective functions.

## Materials and Methods

### Animals

Newborn Sprague-Dawley rat was provided by Beijing Vital River Laboratory Animal Technology Co. Ltd with a license No.SCXK-2017-0005. Animal experiments were carried out in accordance with the Guidelines for Care and Use of Laboratory Animals of Beijing Municipality and approved by Animal Care and Use Committee of Minzu University of China.

### Rat hippocampal neurons

Neurons were isolated and cultured as described previously 12. Hippocampus and cortex of the rat were isolated in DMEM (#03.1006C, EallBio), mechanically dissociated and digested with 0.25% trypsin (#SH30042.01, Hyclone) for 25 min at 37 ^o^C. Cells were centrifuged, resuspended in DMEM containing 10% fetal bovine serum, seeded in poly-L-lysine-coated petri plates and maintained in a humidified air of 5% CO_2_. On the first two days of plating, 10 μM cytosine arabinoside was added to inhibit the growth of glial cells.

### Chemicals

The specific inhibitor of Trk (tyrosine kinase) receptors (TrkB) K252a (#K1639) was purchased from Sigma-Aldrich. Staurosporine (STS, #9953), a broad-spectrum protein kinase inhibitor that was used to induce cell apoptosis, and LY294002 (#9901), a pan-PI3K inhibitor inhibiting PI3K-dependent Akt phosphorylation and signaling was provided by Cell Signaling Technology, Inc. 2,3,5,4′-tetrahydroxystilbene-2-O-β-D-glucoside (THSG, #B21757, HPLC ≥ 98%) was provided by Shanghai yuanye Bio-Technology Co., Ltd.

### MTT cell viability assay and TUNEL cell death assay

Neurons seeded in 96-well plates that were subjected to cell viability assay were incubated with 0.5 mg/mL MTT for 2 h. After discarding the medium, DMSO was added to dissolve the formazan crystals that yield by MTT. The absorption of the solution was measured at 490nm by Epoch 2 microplate spectrophotometer (BioTek Instruments, Inc.).

TUNEL experiments probing death cells were carried out by measuring the 3'-OH of DNA strand breaks. Cell was fixed with 4% paraformaldehyde for 15 min at room temperature, then permeabilized with 0.1% Triton X-100 and 0.1% sodium citrate in 1x PBS for 3 min on ice. TUNEL assay was performed with *in situ* cell death detection kit I (#Roche-11684795910). Cell death rate was obtained from the ratio of TUNEL-positive cells vs DAPI positive cells.

### Immunofluorescence

Neurons seeded on coverslips in 24-well plate were used for experiments. Cells were fixed with 4% paraformaldehyde at room temperature for 15 min, followed by 3 rinses in 1xPBS (1 min each). After incubation with 0.5% Triton X-100 in 1×PBS for 10 min, cells were blocked of the free binding sites with 10% goat serum in 1×PBS at indoor temperature for 1 h, followed by incubation with the primary antibody β-III tubulin (1:500, #T8578, Sigma-Aldrich) or MAP2 (1:500, #4542, Cell Signaling Technology, Inc.) at 4 °C overnight. After rinsing in PBS 3 times, cells were incubated with Alexa Fluor 488-conjugated secondary antibody (Invitrogen) at room temperature for 1.5 h. The nuclei were stained with DAPI (4′, 6-diamidino-2-phenylindole). Cell images were collected using a Leica SP8 confocal microscope.

### Western blot

Proteins of the neurons were extracted by a RIPA lysis buffer (#P0013C, Beyotime Biotechnology) containing 1% phenylmethylsulfonyl fluoride (PMSF, Roche), followed by centrifuging at 4 °C, 13,000 rpm, 25 min to collect the supernatant. Proteins were denatured and separated by 10% SDS-PAGE, transferred to PVDF (polyvinylidene fluoride) membrane. The primary antibodies were used as follow: Anti BDNF (#AF1423), total-Akt (#AF0045), phospho-Akt (Ser473) (#AF1546), Bcl-2 (#AF0060), β-actin (#AF0003) and cleaved-Caspase-3 (#AF1150) were provided by Beyotime Biotechnology. Anti phospho-TrkB (#4619) antibody was provided by Cell Signaling Technology, Inc. The secondary antibodies including: Goat anti-Mouse IRDye 800CW (#926-32210), Goat anti-Rabbit IRDye 800CW (#926-32211), IRDye 680RD Goat anti-Mouse (#926-68070) and IRDye 680RD Goat anti-Rabbit (#926-68071) were provided by Beijing North Yitao Trading Co., Ltd. Western blot images were scanned by the Odyssey CLx infrared fluorescence imaging system (LI-COR Biosciences). The normalized protein expression level was calculated according to the optical densities of the blots that were quantified by Image J software.

### Statistical analysis

Results were presented as means ± S.E.M. Statistical significant difference of variance among the groups were determined by one-way analysis of variance (ANOVA) and followed by Bonferroni's multicomparison tests for *p* value correction in GraphPad Prism 7.0 software.

## Results

### THSG attenuates STS-induced cytotoxicity in hippocampal neurons

**Figure 1:**
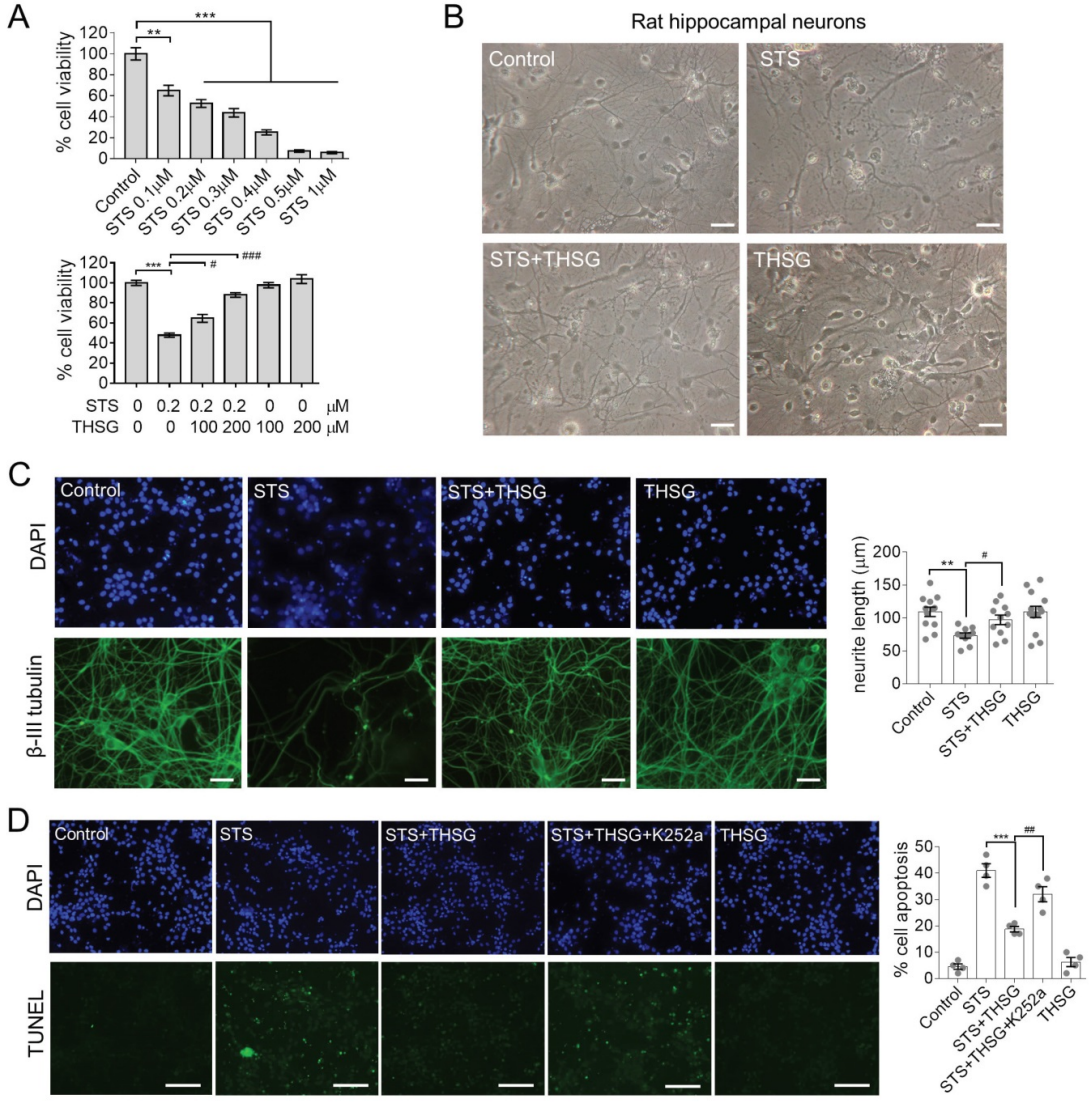
** THSG attenuates STS-induced cytotoxicity in rat hippocampal neurons.** THSG rescued the cell viability of neurons (**A**) and eliminated their morphological collapse from STS-induced cytotoxicity (**B**). (**C**) Immunofluorescent staining of β-III tubulin (a marker protein of neurons) and DAPI (nuclei) suggested the maintenance of neuronal morphology mediated by THSG from STS-induced toxicity. Neurite length was measured by Image J. **, *p<*0.01; #, *p<*0.05. (**D**) TUNEL experiments reported the THSG mediated inhibition of STS-induced apoptosis. However, TrkB inhibitor K252a antagonized the activity of THSG. ***, *p<*0.001. ##, *p<*0.01. (B-D) One-way ANOVA was performed to compare the statistical significance among groups and Bonferroni's multi-comparisons test was used to correct the p value. STS, 0.2 µΜ; THSG, 200 µM. Scale bar, 25 µm.

Hippocampal neurons provide an *in vitro* system for studying neurophysiology, for damage or toxin-induced toxicity in the hippocampus is related to diseases such as encephalitis and AD 11, 12. STS is a natural alkaloid with a broad spectrum inhibitory activity through the prevention of ATP binding to the protein kinase. It is an excellent apoptosis inducer in various cell lines 12, 13. To optimize the concentration to induce toxicity in neurons, STS was administrated to hippocampal neurons at concentrations of 0.1, 0.2, 0.3, 0.4, 0.5 and 1μM for 24 h, followed by MTT assay to assess the cell viability (Figure 1A, upper panel). The cell viability of hippocampal neurons was markedly declined in a dose-dependent manner with STS incubation. Since 0.2 μM STS caused cell death at a rate of 40%-60%, we used it in the following experiments (Figure 1A, lower panel). In the followed experiments, neurons were incubated with THSG for 24 h before STS treatments. The result showed that THSG effectively eliminated STS-induced cytotoxicity and did not exert observed background toxicity in hippocampal neurons (Figure 1A).

**Figure 2:**
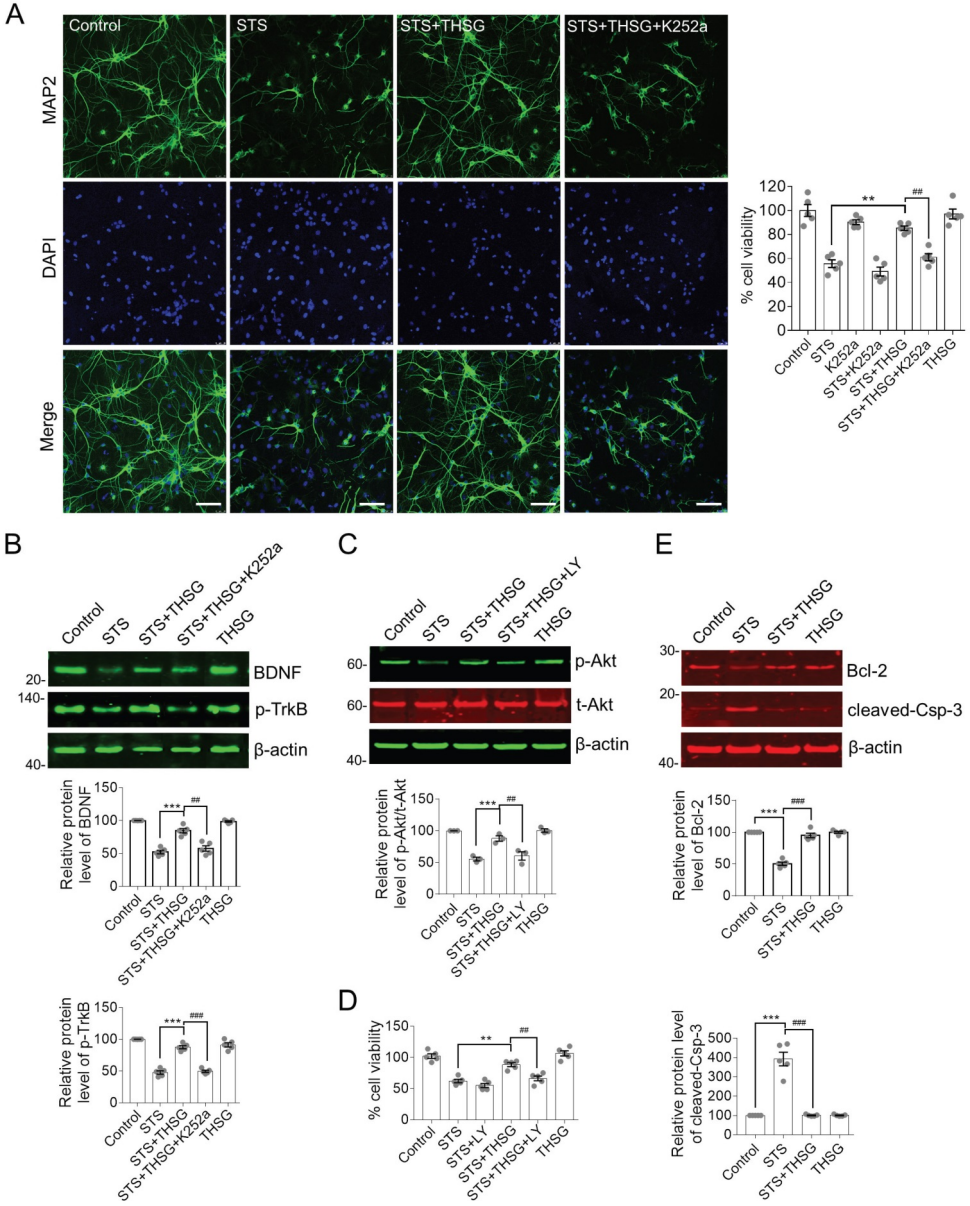
** THSG protects hippocampal neurons from STS-induced cytotoxicity by restoring the biochemistry of BDNF-TrkB/Akt signaling axis and inhibition of apoptosis.** (**A**) THSG maintained the integrity of neuron cell body and neurite outgrowth from STS-induced toxicity. Microtubule-associated protein 2 (MAP2) is a neuron-specific marker. Inhibition of TrkB by K252a (a small inhibitor of TrkB) eliminated the protective effects of THSG. Scale bar, 25 µm. **, *p<*0.01 and ##, *p<*0.01. (**B-C**) THSG restored STS-induced down regulation of BDNF-TrkB and Akt signaling pathway. However, medication of K252a (0.2 µM) or LY294002 (LY, 10 µM, a pan-PI3K/Akt inhibitor) antagonized the activity of THSG. The relative protein expression levels of BDNF, p-TrkB and p-Akt were quantified depending on the density of western blots and normalized to β-actin or t-Akt (for p-Akt), respectively. ** (##), *p<*0.01; *** (###), *p<*0.001. (**D**) Inhibition of TrkB/Akt signaling axis attenuated the protective effect of THSG on cell viability. (**E**) THSG antagonistically restored the expression of Bcl-2 and cleaved-Caspase-3 to alleviate STS-induced apoptosis. (A-E) Statistical significance was examined by one-way ANOVA and followed by Bonferroni's multi-comparisons test to correct *p* value.

Normally, hippocampal neurons showed a slender axon and branch dendrites that interweave to form a neural network, which can be observed by microscopic imaging and immunofluorescence. β-III tubulin is primarily expressed in neurons and widely used as a protein marker to identify neurons. Upon the treatment of STS in an optimized concentration of 0.2 μM, neurons showed significant atrophy of neurite and loss of branching (Figure 1B and C). However, medication of THSG (200 μM) attenuated STS induced cytotoxicity and rescued the morphological decay of neurons. In addition, THSG is nontoxic upon direct administration to neurons (Figure 1B-C), as the morphology of neurons and cell viability in the set of THSG showed identical phenotypes with that of the control set. In addition, THSG mediated attenuation of STS-induced cytotoxicity was confirmed by TUNEL assay (Figure 1D). In STS treated neurons, apoptosis was evoked and produced the break DNA with 3'-OH end that can be probed by TUNEL assay. THSG obviously decreased the rate of cell death from 40% (STS column) to 20% (STS+THSG column). However, the neuroprotective effect of THSG was counteracted by K252a, an efficient inhibitor of tyrosine kinase receptor B (TrkB). Consistently, K252a treatment also counteracted the THSG mediated morphological recovery of hippocampal neurons from STS-induced cellular atrophy, indicated by the immuno-staining of MAP2 and the cell viability assay (Figure 2A). These data suggest that the activity of TrkB is required for THSG mediated neuroprotective effect of hippocampal neurons.

### THSG attenuates STS-induced cytotoxicity by restoring BDNF-TrkB/Akt signaling axis and inhibiting apoptosis

It is interesting to decipher the antagonistic effect of K252a on THSG mediated neuroprotective effects in hippocampal neurons. BDNF is the most extensively studied neurotrophin that is closely associated with neural survival under various pathological conditions. TrkB is a membrane receptor primarily mediated the function of BDNF 10. In view of this, we examined the protein expression of BDNF and TrkB as well as their downstream regulators. In STS-treated hippocampal neurons, BDNF is significantly decreased along with the inhibition of TrkB activity, indicating by the reduced phosphorylation of tyrosine phosphorylation (Figure 2B). Besides, the activity of the cell survival regulator Akt was clearly inhibited (Figure 2C). Akt is the primary effector of BDNF-TrkB signaling axis and its activation is revealed by Ser473 phosphorylation. Consistent with the neuroprotective effect of THSG on hippocampal neurons from STS-induced cytotoxicity, THSG rescued the expression of BDNF coinciding with the reactivation of TrkB and Akt. However, inhibition of TrkB antagonistically attenuated THSG mediated recovery of BDNF expression (Figure 2B, panel of STS+THSG +K252a). In addition, direct inhibition of PI3K/Akt by a specific inhibitor LY294002 (Figure 2C, panel of STS+ THSG+LY) blocked THSG mediated rescue of cell vitality (Figure 2D). Furthermore, under STS-induced cytotoxicity, hippocampal neurons were susceptible to apoptosis as observed by the antagonistically expression of the anti-apoptotic protein Bcl-2 and the apoptosis promoting enzyme cleaved-Caspase-3 (Figure 2E). Similar to the condition of BDNF, THSG restored the expression of Bcl-2 and cleaved-Caspase-3 to inhibit apoptosis and to promote cell survival. From the results as aforementioned, we concluded that STS-induced cytotoxicity impairs the normal morphology of neurons and neurite connectivity and simultaneously down regulates the expression of BDNF and its downstream signaling regulators. THSG effectively restores the expression of BDNF from STS-induced cytotoxicity, reactivates the downstream signaling axis and inhibits apoptosis. BDNF-TrkB and Akt signaling axis is essential for THSG-mediated neuroprotective functions.

**Figure 3:**
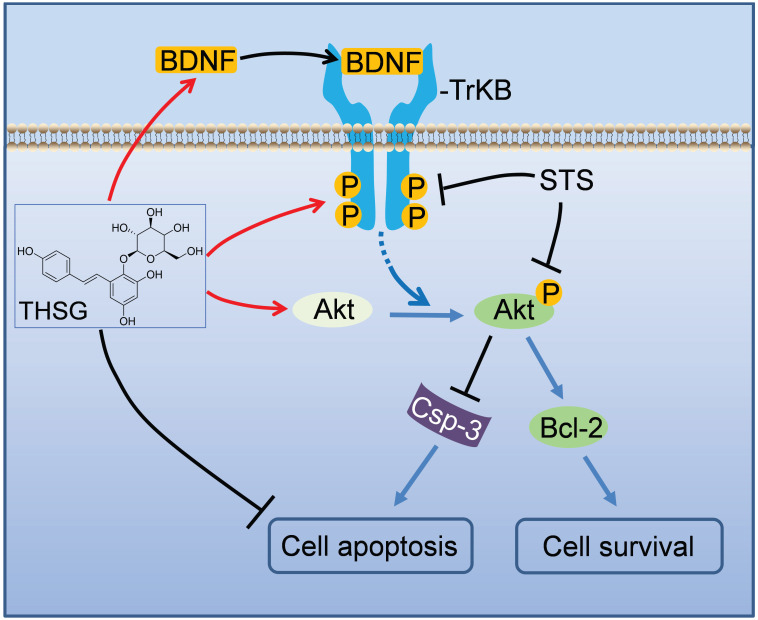
** A schematic model illustrates the neuroprotective effects of THSG on STS-induced toxicity by restoring BNDF-TrkB/Akt signaling axis and inhibiting apoptosis.** In hippocampal neurons, STS down-regulated the expression of BDNF, inhibited TrkB/Akt signaling axis, activated apoptosis-promoting enzyme Caspase-3 and ultimately resulted in neuron apoptosis and death. However, THSG rescued the expression of BDNF, reactivated BDNF-TrkB/Akt signaling pathway, antagonistically restored the biochemistry of Caspase-3 and Bcl-2, and finally promoted cell survival.

In summary, we addressed the neuroprotective effects of THSG in hippocampal neurons (Figure 3). Upon STS induced toxicity, the expression of BDNF was down regulated, along with the inhibition of TrkB and its associated Akt signaling pathway accompanying by initiating apoptosis. THSG attenuated STS-induced toxicity by restoring the expression of BDNF-TrkB and Akt signaling axis and inhibiting apoptosis. Thus, THSG is an excellent neuroprotective agent and BDNF-TrkB signaling axis can be a promise target.

## Discussion

Hippocampal neuron is well-established and widely used to study the neuropathology and screen of drugs. Therefore, we took use of apoptosis inducer staurosporine (STS) to introduce cytotoxicity in cultured neurons, to establish an *in vitro* system for evaluating the neuroprotective effects of THSG. THSG is a characteristic component in *Polygonum multiflorum* with excellent activities of cytoprotection 1, 3, 17. THSG promotes the expression of the longevity gene Klotho and attenuates MPP+-induced neurotoxicity in SH-SY5Y and PC12 cells 1, 7, 18. THSG also prevents chronic restraint stress-induced depression-like behaviors in a mouse model by reducing oxidative stress and inflammatory responses 1, 5. Pharmacokinetic analysis showed that THSG was rapidly absorbed by the gastrointestinal tract and conjugated with α-*D*-glucuronic acid to form intermediate substance and further metabolized. The stilbene moiety may be the effective chemical of THSG to exert neuroprotective function 19. Although THSG is reported to activate signaling pathways like Nrf2/HO-1/NF-κB, BDNF-TrkB, and FGF2-Akt to afford anti-oxidative and anti-inflammation effects 3, 20, how THSG or its metabolites enter or interact with the cell remains unknown. Besides, THSG is not permeable to transport directly through the blood brain barrier. Nevertheless, in neurodegenerative diseases, for instance, THSG ameliorates the cognitive deficits in rodent models of AD and prevents the nigrostriatal degeneration of neurons in MPTP-induced PD models 21, 22. There is compelling evidence accumulated of which supported the regulatory roles of neurotrophins and their related signaling axis in neurodegenerative diseases. Indeed, the typical neurotrophic factor BDNF and its receptor TrkB is essential for neural survival and synaptic activity. Inappropriate expression of BDNF and dysfunctions of its associated signaling axis were frequently observed in a number of neural diseases 8, 10, 23. In light of this statement, we concentrated on the regulation of BDNF and its regulated signaling pathway in THSG mediated neuroprotective functions.

In cultured hippocampal neurons, STS introduced severe toxicity and caused neural collapse, for neurite are vulnerable to toxic insults. The expression of BDNF and the activity of its receptor TrkB were inhibited upon STS treatments (Figure 2B). However, neurons pretreated with THSG successfully maintained their integrity of the cell body and the neurite outgrowth (Figure 1B, C and 2A). Accordingly, the expression of BDNF and TrkB/Akt signaling pathway was rescued and neuronal apoptosis evoked by STS was inhibited, for the expression of Bcl-2 and Caspase-3 was antagonistically restored (Figure 2E). The neuroprotective effect of THSG depends on TrkB/Akt activity, for inhibition of TrkB or Akt associated signaling axis with small inhibitors are capable of eliminating the activity of THSG. These results indicate that the intact TrkB/Akt signaling cascade is essential for THSG to exert neuroprotective effects. This also supports the neurotrophic hypothesis that a positive feedback of BDNF-TrkB signal plays an important role in neural survival 9. Therefore, BDNF-TrkB and Akt signaling axis is a promise target for neuroprotective research and drug discovery.

In summary, we demonstrated the neuroprotective effects of THSG in hippocampal neurons. THSG effectively restores the expression of BDNF and its associated TrkB/Akt signaling axis, to promote neural survival and maintain neurite growth. BDNF-TrkB and their associated signaling regulators may be promising targets for drug medication and disease therapy.

## Abbreviations

BDNF: Brain-derived neurotrophic factor
MTT: 3-(4,5-Dimethylthiazol-2-yl)-2,5-Diphenyltetrazolium Bromide
STS: staurosporine
MPP+: 1-methyl-4-phenylpyridinium
THSG: 2,3,5,4′-Tetrahydroxystilbene-2-O-β-d-glucoside
TrkB: Trk (tyrosine kinase) receptor B, also known as Tropomyosin receptor kinase B
TUNEL: terminal deoxynucleotidyl transferase mediated dUTP nick-end labeling
